# Mitochondrial metabolism regulation and epigenetics in hypoxia

**DOI:** 10.3389/fphys.2024.1393232

**Published:** 2024-06-10

**Authors:** Madison Laird, Jennifer C. Ku, Jacob Raiten, Sashwat Sriram, Megan Moore, Yong Li

**Affiliations:** ^1^ Western Michigan University Homer Stryker School of Medicine, Kalamazoo, MI, United States; ^2^ Department of Orthopaedic Surgery, Biomedical Engineering, Western Michigan University Homer Stryker School of Medicine, Kalamazoo, MI, United States

**Keywords:** mitochondrial metabolism, epigenetic modifications, hypoxia, gene expression, oxygen

## Abstract

The complex and dynamic interaction between cellular energy control and gene expression modulation is shown by the intersection between mitochondrial metabolism and epigenetics in hypoxic environments. Poor oxygen delivery to tissues, or hypoxia, is a basic physiological stressor that sets off a series of reactions in cells to adapt and endure oxygen-starved environments. Often called the “powerhouse of the cell,” mitochondria are essential to cellular metabolism, especially regarding producing energy through oxidative phosphorylation. The cellular response to hypoxia entails a change in mitochondrial metabolism to improve survival, including epigenetic modifications that control gene expression without altering the underlying genome. By altering the expression of genes involved in angiogenesis, cell survival, and metabolism, these epigenetic modifications help cells adapt to hypoxia. The sophisticated interplay between mitochondrial metabolism and epigenetics in hypoxia is highlighted by several important points, which have been summarized in the current article. Deciphering the relationship between mitochondrial metabolism and epigenetics during hypoxia is essential to understanding the molecular processes that regulate cellular adaptation to reduced oxygen concentrations.

## Introduction

Widely referred to as the “powerhouse of the cell,” mitochondria utilize the process of oxidative phosphorylation to produce ATP, fueling basic physiological functions of the cell, including growth, movement, and homeostasis. In addition to their role in carbon metabolism, mitochondria also play critical physiological roles in regulating apoptosis (Wang and Youle, 2009), inflammation (Marchi et al., 2023), reactive oxygen species production (Zorov et al., 2014), calcium signaling (Rizzuto et al., 2012)^,^ and steroid synthesis. (Miller, 2013). Consequently, mitochondrial dysfunction has been associated with a range of human diseases, including neurodegenerative disorders, cardiovascular diseases, diabetes, and certain types of cancer. (Lin and Beal, 2006; Sivitz and Yorek, 2010; Poznyak et al., 2020).

Derived from an alphaproteobacterial endosymbiont, mitochondria are ubiquitous eukaryotic organelles composed of two distinct membranes that encapsulate an intermembrane space and matrix (Gray et al., 1979). Mitochondria contain their own maternally inherited genome of circular, double-stranded DNA (mtDNA) that is packaged into nucleoids (Kukat et al., 2011; Chinnery and Hudson, 2013). The mammalian mitochondrial genome encodes 13 proteins, 22 transfer RNAs, and 2 ribosomal RNAs. Both mtDNA and nuclear DNA contribute to the development of the electron transport chain (ETC), which drives oxidative phosphorylation (Habbane et al., 2021). Within the ETC, electrons participate in a sequence of redox reactions, releasing energy in the form of a proton gradient. Essential to normal cell function, oxygen serves as the final electron acceptor in the ETC, allowing the energy from the proton motive force to be harnessed by ATP-synthase to produce ATP (Nolfi-Donegan et al., 2020).

While oxygen is required by all mammalian cells, different tissues are exposed to varying concentrations under physiological (i.e., physioxic) conditions, reflecting differences in metabolic demands (Ncbi, 2024). Within peripheral tissues, levels of oxygen correlate to a balance between supply and demand, ensuring proper physiological function while avoiding oxygen toxicity or hypoxia (i.e., a state of insufficient oxygen delivery) (Pias, 2020). For instance, interaction of lung tissue with atmospheric air during respiration exposes alveoli to about 13.5% oxygen (translating to a partial pressure of roughly 100 mm Hg), though these values can change depending on factors such as altitude and respiratory status (McKeown, 2014). Alternatively, in the adult human brain, physioxic oxygen levels can range between 11 and 53 mmHg depending on the region, with levels below 10 mmHg considered hypoxic with resulting impairment in ATP production (Zhang K. et al., 2011). Similarly, in skeletal muscle, oxygen concentration generally varies between 15 and 76 mmHg depending on location. When oxygen levels within skeletal muscles fall below 15 mmHg, the tissue is considered hypoxic and becomes increasingly reliant on adaptive mechanisms to maintain viability (Pircher et al., 2021).

In hypoxia, cells cannot meet their energy demands through oxidative phosphorylation. Adaptive mechanisms are triggered within mammalian cells to promote cell survival and reestablish a normoxic environment (Paffett and Walker, 2007). Such mechanisms are regulated primarily by hypoxia-inducible factors (HIFs)—heterodimeric transcription factors that influence expression of genes meant to maintain cellular homeostasis and increase oxygen delivery to deprived tissues (Choudhry and Harris, 2018). HIFs are composed of an unstable α-subunit paired with a constitutively expressed HIF-β subunit (also known as aryl-hydrocarbon receptor nuclear translocator, ARNT) (Sousa Fialho et al., 2019). Three isoforms of α-subunit exist: HIF1-α, HIF2-α, and HIF3-α. While each regulates a unique catalog of genes, all α-subunits are collectively under the control of various proteins known as α-ketoglutarate-dependent dioxygenases. One example of such proteins is members of the prolyl hydroxylase domain (PHD) family (Weidemann and Johnson, 2008). During periods of sufficient oxygen delivery, diatomic oxygen is recognized by these prolyl hydroxylase enzymes, which respond as their name suggests by hydroxylating conserved proline residues within HIF-α subunits (Wong et al., 2013). Hydroxylation of HIF-α proline residues creates a binding site for the tumor-suppressor protein von Hippel-Lindau (VHL)—a polyubiquitin ligase which functions to ubiquitinate α-subunits and mark them for proteasomal degradation (Li and Kim, 2011). Conversely, when oxygen concentrations are insufficient to meet cellular metabolic demands, PHD proteins are inactive, leaving α-subunits free to accumulate and dimerize with HIF-β monomers (Ziello et al., 2007). HIF-α subunits are also subject to regulation by another class of oxygen-dependent enzymes known as asparaginyl hydroxylases, specifically Factor Inhibiting HIF (FIH-1) (Sim et al., 2018). While PHDs primarily target proline residues on HIF-α subunits for hydroxylation, FIH-1 instead hydroxylates asparagine residues within the HIF-α C-terminal transactivation domain (CTAD) (Lando et al., 2002). Such hydroxylation events inhibit interactions between HIF-α and necessary transcriptional co-activators p300/CBP, thereby serving to negatively regulate HIF transcriptional activity in the presence of physioxic conditions. Alternatively, if oxygen concentrations are below threshold levels, FIH-1 cannot carry out hydroxylation reactions and HIF-α subunits are allowed to accumulate and interact with HIF-β and impact gene expression (Zhang et al., 2010). The completed HIF cytosolic complex is then translocated to the nucleus where it interacts with specific DNA domains known as hypoxia-response elements (HREs) (Kaluz et al., 2008). Interaction with HIFs at these loci activates transcription of a diverse array of genes (some reviews estimating upwards of 200), earning them the moniker of “master regulators” of oxygen homeostasis (Denko et al., 2003).

In addition to oxygen, PHD and FIH-1 enzymes (and thus HIF activity) are also regulated by intermediates of the tricarboxylic acid cycle (TCA cycle, also known as the citric acid cycle or Kreb’s cycle) and reactive oxygen species (ROS) (Hagen, 2012; Bailey and Nathan, 2018). In particular, TCA intermediates succinate and fumarate can inhibit hydroxylation by these enzymes by competing with α-ketoglutarate and interfering with its function as a required co-substrate (Avgustinova and Benitah, 2016). Ultimately, such interactions result in stabilization of HIF-α subunits and formation of HIF transcriptional complexes, even under normoxic conditions—a phenomenon known as pseudohypoxia (Hayashi et al., 2019). However, the effects of ROS on hydroxylase function are more nuanced and may differ depending on the levels of oxidative stress. For instance, moderate levels of ROS can stimulate PHD and FIH-1 activity, thus promoting hydroxylation of HIF-α and ultimately dampening HIF transcriptional effects. However, when cells are under significant oxidative stress, the resulting high levels of ROS production serve to interfere with PHD and FIH-1 activity, thereby stabilizing HIF-α and increasing transcription of HIF-controlled genes (Fong and Takeda, 2008). Such high levels of TCA intermediates and ROS with resulting PHD/FIH-1 dysregulation is seen in periods of metabolic stress and have been implicated in various pathological conditions, including cancer, inflammation, and ischemic diseases.

The range of cellular functions affected by HIF signaling pathways is indeed vast, all culminating in a response that hardens cells to the hypoxic environment in which they find themselves (Luo et al., 2022). Briefly, HIF activation exerts considerable influence over the body’s oxygen delivery capacity by promoting angiogenesis and erythropoiesis, while also modulating cellular oxygen utilization and progression through the cell cycle (Krock et al., 2011; Haase, 2013; Ortmann et al., 2014; Thomas and Ashcroft, 2019).

As the major consumers of oxygen within cells, mitochondria are exquisitely sensitive to decreased oxygen availability (Fuhrmann and Brüne, 2017). Importantly, hypoxia and HIF signaling also control mitochondrial function through the modification of organelle morphology, metabolic activity, and reactive oxygen species (ROS) formation (Huang et al., 2022). Such mechanisms are critical for maintaining cell viability during periods of physiological hypoxia (e.g., altitude changes, physical exertion) and promoting a return to homeostatic conditions (Kumar and Choi, 2015; Taylor and Scholz, 2022). However, these same pathways may also be conscripted in various disease states and utilized in ways that promote pathogenesis and disease progression (Park et al., 2004; Lee et al., 2019). Accordingly, a better understanding of the physiologic response to hypoxia can uncover novel therapeutic approaches for a variety of morbid conditions including cancer, diabetes, and inflammatory disorders. In this review, we discuss hypoxia signaling pathways within mitochondria, highlighting the significance of these pathways and their impact on the epigenome.

## Hypoxia signaling and mitochondrial function

As was discussed above, HIF is the primary effector of the cellular response to hypoxia, and HIF-mediated changes in gene expression have a profound impact on many facets of mitochondrial function, particularly as it relates to energy metabolism (Li et al., 2022).

**FIGURE 1:**
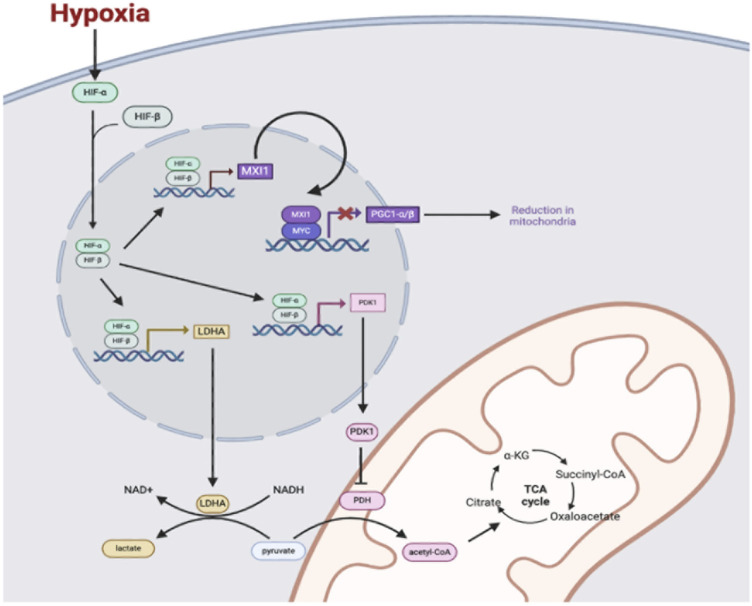
Under hypoxic conditions, HIF-α is stabilized and dimerizes with HIF-β. The HIF-α and HIF-β complex enters the nucleus and binds to hypoxia response elements (HREs) to transcriptionally activate genes including genes coding for the proteins pyruvate dehydrogenase 1 (PDK1), lactate dehydrogenase A (LDHA), and MAX interaction 1 (MXI1). PDK1 enters the cytosol to phosphorylate and inhibit pyruvate dehydrogenase (PDH), which decreases pyruvate conversion to acetyl-CoA and decreases flux through the TCA cycle in mitochondria. LDHA converts pyruvate in the cytosol to lactate to regenerate NAD+ to fuel glycolysis for the generation of ATP in anaerobic conditions. Additionally, the HIF-1 and HIF-β complex upregulates the transcription of MAX interactor 1 (MXI1), which interacts with Myc protein to repress expression of the gene for peroxisome proliferation-activated receptor-gamma coactivator 1α and 1β (PGC1 α/β). Reduction of PGC1 α/β leads to a reduction in mitochondria.

As one example, HIF signaling induces the suppression of oxidative phosphorylation and a transition towards anaerobic glycolysis, allowing cells to continually meet energy demands while simultaneously reducing oxygen requirements (Li et al., 2019). Under normoxic conditions, glycolysis results in the generation of pyruvate, which is ultimately transported into the mitochondria and converted to acetyl-CoA via the enzyme pyruvate dehydrogenase (PDH) (Naifeh et al., 2023). In the process, the reduced cofactor NADH is also generated. (Chaudhry, 2023). Once acetyl-CoA has been produced within mitochondria, it enters the TCA cycle where it can be used to generate additional reduced cofactors (Alabduladhem, 2022). The electrons in NADH and FADH2 are then fed into the mitochondrial ETC, wherein a series of protein complexes utilize the energy to establish a proton motive force (Cooper, 2000). As electrons pass through the ETC complexes, some prematurely combine with diatomic oxygen and result in the production of ROS (i.e., superoxide, hydrogen peroxide, and hydroxyl radical) that have the potential to damage mitochondrial and extra-mitochondrial macromolecules (Zhao et al., 2019). Passage of protons through membrane-bound ATP synthase complex and back into the mitochondrial matrix then allows for the generation of ATP, which completes the process of oxidative phosphorylation (Neupane et al., 2019). However, during periods of hypoxia, HIF-1 induces the expression of pyruvate dehydrogenase kinase 1 (PDK1), which functions to inhibit PDH and prevent the conversion of pyruvate into acetyl-CoA (Kim et al., 2006). Decreased acetyl-CoA reduces flux through the TCA cycle, which ultimately results in decreased generation of reduced cofactors NADH and FADH_2_, decreased flux through the ETC, and consequently, decreased oxygen consumption (Kim et al., 2006; Martínez-Reyes and Chandel, 2020; Taylor and Scholz, 2022). At the same time, HIF-1 upregulates expression of the enzyme lactate dehydrogenase A (LDHA), which allows for the reduction of accumulating cytosolic pyruvate into lactate (Cui et al., 2017). The importance of this process is that it allows for the regeneration of oxidized NAD^+^, which can then be used in repetitive cycles of glycolysis to allow for continued ATP generation via anaerobic mechanisms (Spinicci et al., 2022). To further enhance this shift in metabolism, HIF-1 also acts to upregulate expression of all enzymes in the glycolytic pathway, as well as glucose transporters GLUT1 and GLUT3 in order to increase cellular glucose uptake and maintain sufficient levels of ATP production (Kierans and Taylor, 2021; Chang et al., 2022). In summary, the HIF-mediated shift in cellular metabolism away from aerobic respiration significantly reduces oxygen demand, while also reducing mitochondrial ROS production and protecting the cell against radical-mediated damage (Samanta and Semenza, 2017) (Figure 1).

Furthermore, HIF-1 impacts mitochondrial bioenergetics via its influence over LON protease and cytochrome c oxidase subunit 4 (Semenza, 2011; Voos and Pollecker, 2020). LON protease is a mitochondrial ATP-dependent protease that normally functions to degrade misfolded proteins and maintain mitochondrial protein quality during periods of oxidative stress (Voos and Pollecker, 2020). When oxygen levels are low, HIF-1 upregulates expression of the LON protease gene to help block inappropriate buildup of damaged proteins and ensure proper mitochondrial function and energy production under hypoxic conditions (Douiev et al., 2021). Cytochrome c oxidase (COX), also known as complex IV, is a key enzyme complex in the mitochondrial ETC responsible for the final step of aerobic respiration (i.e., reduction of oxygen to water.) COX4 is a regulatory subunit of this complex with two distinct isoforms, COX4-1 and COX4-2, both of which are regulated by HIF-1 (Douiev et al., 2021). Under hypoxic conditions, HIF-1 activation leads to increased expression of COX4-2 and decreased expression of COX4-1 (via LON protease-mediated degradation), helping to optimize mitochondrial function under low oxygen conditions (Bao et al., 2021). For example, upregulation of COX4-2 may enhance efficiency of oxygen utilization by the ETC due to its higher affinity for oxygen compared to COX4-1, allowing for relatively lower concentrations of oxygen to produce sufficient levels of ATP (Bao et al., 2021). In tandem, downregulation of COX4-1 expression may reflect an adaptive response to prioritize COX4-2 synthesis and optimize mitochondrial bioenergetics in low-oxygen environments (Huang et al., 2022).

In addition to changes in metabolism, HIFs also function to regulate levels of mitochondria within cells via influence over several mitochondrial homeostatic mechanisms (Huang et al., 2022). Together, these actions serve to prevent ROS production by mitochondria, and thus limit the potential for further cellular damage during periods of hypoxia (Špaková et al., 2021). For instance, HIF-1 has been shown to decrease mitochondrial biogenesis through the transcriptional activation of MAX interactor 1 (MXI1) (Corn et al., 2005; Löfstedt et al., 2009). This increase in MXI1 causes MYC to form a dimer with MXI1. The MXI1-MYC complex then binds to E-box elements in the nucleus and blocks the promoter region for peroxisome proliferator-activated receptor-γ coactivator 1α and 1β (PGC1α/β), thus expression of PGC1α/β is reduced (O'Hagan et al., 2009; LaGory et al., 2015). Whereas normally, PGC1α/β acts to increase mitochondrial biogenesis, a decrease in protein levels leads to a reduction in mitochondrial mass with a concurrent reduction in ROS generation. Moreover, hypoxia, via HIF-1 signaling, induces mitochondrial autophagy (also known as mitophagy) in a process requiring HIF-1-dependent expression of BCL2-interacting protein 3 (BNIP3) (Zhang et al., 2019; Halling and Pilegaard, 2020; Zhao et al., 2020). Interestingly, while BNIP3 is normally a pro-apoptotic mediator, Bellot et al. reported that its activation by HIF-1 and subsequent induction of mitophagy can limit ROS production and provide alternative sources of nutrients and energy during hypoxic conditions (Bellot et al., 2009). Upregulation of mitophagy recycles damaged mitochondria that would otherwise trigger apoptosis, thus the balance between apoptosis and mitophagy shifts away from apoptosis and cell survival is increased.

Finally, HIF regulated gene expression has been shown to influence mitochondrial dynamics, primarily via the promotion of fission and inhibition of mitochondrial fusion. Fission—the process of mitochondrial segmentation into smaller discrete organelles—is also implicated in the promotion of mitophagy (Burman et al., 2017; Chen et al., 2019). The primary protein regulating mitochondrial fission is dynamin related protein 1 (DRP1), a cytosolic GTPase whose expression is directly upregulated by HIF-1 (Matilainen et al., 2017). The upregulation of fission by HIF-1 during periods of hypoxia suggests this process could serve as a protective mechanism against hypoxia-induced mitochondrial damage, in addition to limiting aerobic respiratory capacity and ROS production.

### Mitochondrial metabolism and epigenetic modifications

While it is evident that mitochondria play a crucial role in glucose and lipid metabolism, recent studies over the last decade have revealed roles beyond cellular energy production. Normal mitochondrial function is essential for providing key intermediate metabolites necessary for epigenetic modifications within the nuclear genome, such as DNA and histone modifications (Matilainen et al., 2017). These modifications regulate gene expression through alterations in chromatin structure and/or by modulating the access of regulatory proteins and transcription machinery (Miller and Grant, 2013). As described above, hypoxic conditions have a profound effect on mitochondrial metabolism, thereby altering the levels of the key metabolites that function within this mitochondria-nuclear crosstalk (Lopes A, 2020; Wiese and Bannister, 2020).

**FIGURE 2:**
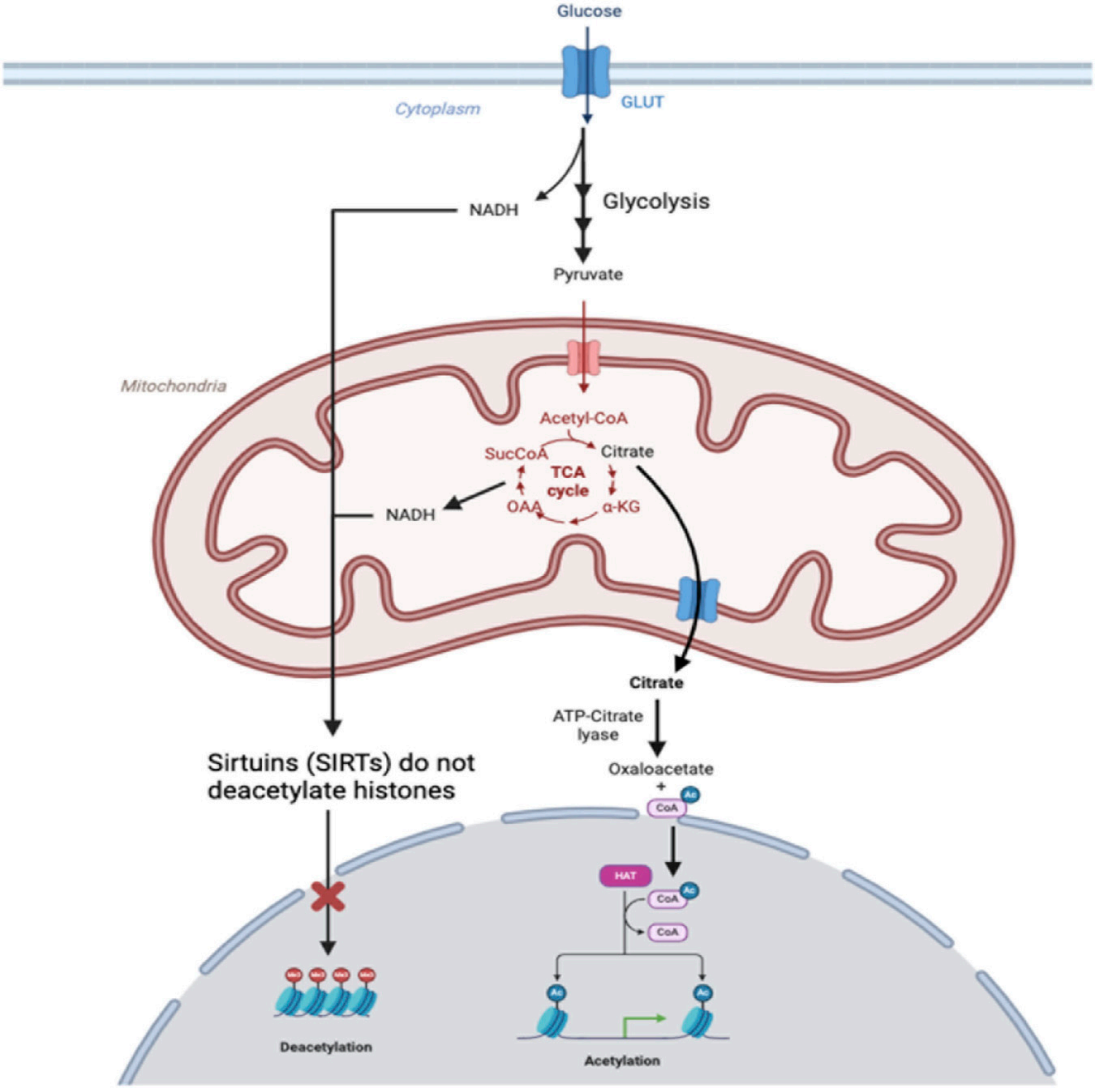
In hypoxic conditions, glucose imported into cells through glucose transporters (GLUTs) is used to fuel anaerobic metabolism, leading to the production of NADH in glycolysis and accumulation of NADH from the TCA cycle that is not used to fuel oxidative phosphorylation. Production of NADH depletes NAD+, which is a substrate required for sirtuins (SIRTs) to deacetylase histones, so histones remain acetylated. Citrate produced in mitochondria is exported.

For example, α-ketoglutarate and acetyl-CoA generated through mitochondrial metabolism have been observed to influence histone modification. Within the mitochondrial matrix, citrate synthase converts acetyl-CoA and oxaloacetate into citrate. Under a high-energy state, the mitochondrial citrate carrier (CiC) within the inner mitochondrial membrane exports citrate into the cytosol. There, in the presence of ATP and CoA, it is subsequently metabolized to acetyl-CoA and oxaloacetate via ATP-citrate lyase (ACLY) (Figure 2). The extramitochondrial acetyl-CoA produced not only supports lipogenesis but also serves as a key acetyl group donor for histone acetylation by histone acetyltransferases (HAT) (Wellen et al., 2009; Tarazona and Pourquié, 2020; Mosaoa et al., 2021). While not fully understood, the loosening of chromatin structure and the subsequent increase in gene expression through histone acetylation are believed to enhance the availability of TCA intermediates, such as α-ketoglutarate (α-KG), which is an important cofactor for histone demethylases (Gao et al., 2018). While CiC expression and activity are primarily governed by intracellular energy states, hypoxia has been shown to play a role in upregulating CiC gene expression. Under hypoxia, CiC expression is mediated by the stabilization and activation of HIF-1α. HIF-1α binds to the promoter region of the CiC gene, promoting its transcription, thereby enhancing the transport of citrate from mitochondria to cytosol to support metabolic adaptations in low oxygen conditions. Increased cytosolic citrate results in elevated acetyl-CoA levels, contributing to increased histone acetylation. Conversely, under normoxia, CiC expression is typically lower due to the absence of HIF-1α activation, resulting in reduced citrate transport activity and decreased histone acetylation (Mosaoa et al., 2021). For example, a study investigating the role of oxygen levels in mesenchymal stem cell differentiation found that under normoxic conditions, the activity of CiC is impaired, resulting in elevated acetyl-CoA levels within the mitochondria. The accumulation of acetyl-CoA in the mitochondria then increases oxidative metabolism and decreases histone acetylation. However, under hypoxic conditions, CiC activity was upregulated, resulting in increased export of acetyl-CoA and subsequent histone acetylation, thus allowing for mesenchymal stem cell differentiation (Morganti et al., 2020; Pouikli et al., 2022). In a different study, exposure to acute or chronic cycling of hypoxia also resulted in increased expression of CIC in lung cancer, prostate, and glioblastoma cells (Hlouschek et al., 2018).

Sirtuins (SIRT), members of class III histone deacetylases, have also been implicated as important regulators of histone acetylation (Grabowska et al., 2017; Lisowski et al., 2018; Pecher et al., 2020). Mammals have 7 SIRT proteins, namely, SIRT1 through SIRT7, each with its own specialized function and subcellular localization. SIRT1, SIRT6, and SIRT7 are primarily located in the nucleus, while SIRT3, SIRT4, and SIRT5 reside in mitochondria, and SIRT2 predominantly occupies the cytoplasmic and nuclear compartments (Turrens, 2003). These sirtuins are exposed to different pools of NAD+/NADH depending on their subcellular localization. The SIRTs predominantly found in the nucleus—SIRT1, SIRT6, and SIRT7—are activated by an increase in the NAD+/NADH ratio because SIRTs use NAD + as a substrate. Importantly, these SIRTs are histone deacetylases (HDACs) that transfer acetyl groups of lysine residues on histones to NAD+ (Garofalo et al., 1988; Yun et al., 2012; Gut and Verdin, 2013; Lee, 2019). Under hypoxic conditions, mitochondrial oxidative metabolism is inhibited, resulting in a decrease in NAD+, which primarily affects the activity of mitochondrial SIRT3, SIRT4, and SIRT5, leading to altered mitochondrial metabolism and antioxidant activity. This decrease in NAD + leads to decreased SIRT activity and decreased histone deacetylation within the nucleus, along with concomitant histone hyperacetylation, thus resulting in unregulated DNA transcription. Under normoxia, the NAD+/NADH ratio remains adequate for SIRTs located in the nucleus to exert their regulatory effects on histone acetylation.

As discussed, the electron transport chain (ETC) on the inner membrane of mitochondria facilitates the production of adenosine triphosphate (ATP) through a series of electron-transfer reactions that pump protons into the mitochondrial intermembrane space to create a driving force for ATP synthase to produce ATP (Turrens, 2003). Generally, oxygen serves as the final electron acceptor in the ETC but oxygen can also be prematurely reduced by electrons in the ETC to form superoxide anions (O2⋅−). Superoxide is classified as a reactive oxygen species (ROS) because it contains an unpaired electron known as a free radical, making it unstable and reactive. Superoxide is also a precursor for many other ROS and can be converted to hydrogen peroxide (H2O2) spontaneously or in a reaction catalyzed by enzymes in the superoxide dismutase family. ROS may be beneficial at moderate levels to play a role in cellular signaling, but an excess of ROS drives the oxidation of macromolecules leading to cell damage (Dröge, 2002). Through several mechanisms, ROS can also modify DNA bases and histones, and therefore alter the epigenome of a cell (Kietzmann et al., 2017).

Although hyperoxia corresponds to an increase in ROS production due to the availability of excess oxygen for premature reduction, hypoxia has also been found to promote increased ROS production (Guzy et al., 2005). While many intracellular sites contribute to ROS generation, mitochondria are recognized as a principal source (Sies and Jones, 2020). Specifically, complex I plays a significant role in sensing acute hypoxia and is known for its high capacity to produce ROS, primarily generating superoxide and hydrogen peroxide (Duong et al., 2021; Okoye et al., 2023). Interestingly, the activity of complex I has been shown to be regulated by reversible oxidation, thereby providing an intervention to attenuate the adverse effects of hypoxia (Onukwufor et al., 2022).

Furthermore, under hypoxic conditions, the quinone-binding sites (Qi and Qo) of complex III, are critical sites of increased superoxide generation. In complex III, production of superoxide at the Qo site enhances the release of superoxide into the intermembrane space where it can diffuse to the cytosol (Chen et al., 2003). The superoxide dismutases found in the intermembrane space and cytosol contain copper and zinc in their active sites. Alternatively, ROS in the intermembrane space can be converted to less reactive forms by reducing cytochrome c and returning the extra electron to the ETC. Superoxide produced at the Qi site of complex III is released into the mitochondrial matrix (Chen et al., 2003). These ROS can be neutralized by the form of superoxide dismutase expressed in the matrix, which contains manganese as the active metal. The manganese-containing superoxide dismutase is upregulated in response to various cellular stressors such as increased oxygen concentration (Fridovich, 1995).

Although excessive ROS production in hypoxia can lead to cell death, sublethal levels can offer protective benefits by inhibiting prolyl hydroxylase domain (PDH), the inhibitor of HIF-1a and thus upregulating HIF-1a levels. (Tafani et al., 2016). Increased HIF-1a leads to upregulation of proteins that facilitate angiogenesis, proliferation, and stem cell renewal. (Tafani et al., 2016).

### Mitochondrial metabolism and epigenetic reprogramming

Reactive oxygen species (ROS), as highly reactive oxygen-containing intermediates, also have considerable influence over the delicate equilibrium of stem cell potency, reprogramming, and mitochondrial function. Maintaining STEM cell pluripotency relies on the precise regulation of ROS levels, encompassing superoxide radical anion (O2⋅ −), hydroxyl radical (⋅OH), hydrogen peroxide (H2O2), and lipid hydroperoxides (LOOH) (Ivanova and Lyublinskaya, 2021). Low ROS levels play a pivotal role in preserving genome integrity and preventing differentiation (Tan and Suda, 2018; Chakrabarty and Chandel, 2021). Mitochondrial ROS generation inhibition becomes imperative for sustained pluripotency, orchestrated by key players such as superoxide dismutase 1 and 2 (SOD1/2) strategically positioned in the mitochondrial intermembrane space and matrix. Peroxiredoxins further modulate H2O2 levels (Palma et al., 2020). In addition, transcription factors, including the Forkhead Box Protein O (FOXO) family and nuclear factor (erythroid-derived 2)-like 2 (NRF2), intricately govern the expression of antioxidant genes (Liang and Ghaffari, 2018; Tan and Suda, 2018). Intriguingly, inhibition of the mitochondrial electron transport chain during STEM cell reprogramming towards glycolytic metabolism is associated with a reduction in ROS (Jaworska et al., 2023). Conversely, mutations in mitochondrial DNA (mtDNA) mark a significant increase in ROS generation, impacting the efficiency of pluripotent stem cell reprogramming and self-renewal capacity (Hämäläinen et al., 2015).

The profound impact of mitochondrial metabolism on epigenetic reprogramming and cell-fate decisions unfolds through the lens of the 2-oxoglutarate-dependent dioxygenase (2OGDDs) superfamily. Comprising Jumonji C-domain-containing histone demethylases (JHD) and ten–eleven translocation (TET) methylcytosine dioxygenase enzymes, this superfamily orchestrates histone and DNA demethylation processes (Bargiela et al., 2018). The TET family, particularly TETs 1-3, assumes a pivotal role in converting 5-methylcytosine (5-mC) to 5-hydroxymethylcytosine (5-hmC), a demethylating base (Lio and Rao, 2019). Under hypoxia, TET1 and TET2 activity is upregulated, leading to increased transcription of glycolytic genes (Aljoufi et al., 2022). Mitochondrial dysfunction and the accumulation of succinate and fumarate, however, inhibit TET activity, resulting in reduced 5-hmC levels (Laukka et al., 2016). JHDs also act as α-ketoglutarate-dependent mechanisms for cytosine demethylation, face inhibition from succinate and fumarate, further underscoring the nuanced regulatory network governing STEM cell fate (Avgustinova and Benitah, 2016; Ly et al., 2020). Additionally, pyruvate metabolism and regulation also play a role in the maintenance of pluripotency and preventing differentiation in stem cells. Uncoupling protein 2 (UCP2) is upregulated in pluripotent STEM cells and is responsible for shunting pyruvate away from mitochondria, associated with the shift towards glycolytic metabolism (Zhang J. et al., 2011). During stem cell differentiation, however, UCP2 is downregulated (Rupprecht et al., 2019).

### Summary

In hypoxic conditions, the junction of mitochondrial metabolism and epigenetics demonstrates the multifaceted and dynamic interconnection between cellular energy regulation and gene expression modification. Hypoxia is a fundamental physiological stressor that triggers a series of cellular events to adapt and survive under oxygen-starved conditions. To increase survival, cells respond to hypoxia by altering their metabolism. Mitochondrial metabolic intermediates are critical to epigenetic modifications in the nuclear genome, regulating gene expression by modifying the structure of DNA and histone proteins without altering the underlying genetic instructions. Therefore, under hypoxic conditions, mitochondrial metabolism is linked to epigenetic changes. Moreover, hypoxia also impacts reactive oxygen species (ROS) concentrations, the formation of which is significantly regulated by mitochondria, subsequently influencing changes in the epigenome. These epigenetic changes aid in the adaptation of cells to hypoxia by modifying the expression of genes related to angiogenesis, cell survival, and metabolism. This article provides a summary of the main ideas that emphasize the intricate relationship between mitochondrial metabolism and epigenetics in hypoxia.

## Discussion and future direction

The complex relationship between cellular energy regulation and gene expression modulation is exemplified by the intersection of mitochondrial metabolism and epigenetics in hypoxic environments. During hypoxia, cells are unable to meet their energy requirements through oxidative phosphorylation, prompting the activation of adaptive mechanisms largely governed by hypoxia-inducible factors (HIFs). These adaptive responses, orchestrated by a variety of HIF-induced signaling pathways, ultimately aim to preserve cellular homeostasis under unfavorable conditions. Hypoxia and HIF signaling also exert control over mitochondria, which are major consumers of oxygen within the cell, by modifying metabolic pathways, ROS production, and organelle morphology.

As summarized in this article, the downstream effects of hypoxia are linked to epigenetic changes within the cell, highlighting the role of mitochondria as epigenetic regulators in both normoxic and hypoxic conditions. Understanding the relationship between mitochondrial metabolism and epigenetics is crucial for deciphering the molecular mechanisms underlying cellular adaptation to reduced oxygen concentrations. Moreover, it holds significant implications for various fields, including aging, ischemic disease, and cancer.

In conclusion, continued research into the crosstalk between mitochondrial metabolism and epigenetics in hypoxic environments will shed light on fundamental cellular processes and may lead to the development of novel therapeutic strategies for conditions influenced by hypoxia.
